# Psychometric evaluation of the Persian version of the Sense of Belonging in Nursing School (SBNS): a quantitative and cross-sectional design

**DOI:** 10.1186/s12912-024-01738-x

**Published:** 2024-01-27

**Authors:** Seyedmohammad Mirhosseini, Hamid Sharif-Nia, Maede Esmaeili, Fatemeh Ameri, Hamed Khosravi, Ali Abbasi, Hossein Ebrahimi

**Affiliations:** 1 Department of Nursing, School of Nursing and Midwifery, Shahroud University of Medical Sciences, Shahroud, Iran; 2 Psychosomatic Research Center, Mazandaran University of Medical Sciences, Sari, Iran; 3 Department of Nursing, Amol Faculty of Nursing and Midwifery, Mazandaran University of Medical Sciences, Sari, Iran; 4 Student Research Committee, School of Nursing and Midwifery, Shahroud University of Medical Sciences, Shahroud, Iran; 5 Nursing Care Research Center, Semnan University of Medical Sciences, Semnan, Iran; 6 Center for Health Related Social and Behavioral Sciences Research, Shahroud University of Medical Sciences, Shahroud, Iran

**Keywords:** Sense of belonging, Nursing, Validity, Reliability, Psychometric

## Abstract

**Background:**

This study aimed to evaluate the psychometric indicators of the Persian version of the Sense of Belonging in Nursing School scale (SBNS).

**Methods:**

The study conducted in Shahroud and Semnan schools of nursing and midwifery in Iran examined nursing students using a cross-sectional approach by convenience sampling method from 3/6/2023 to 24/8/2023. To assess the SBNS scale, the forward–backward procedure was used to translate it into Persian. Face and content validity were evaluated, and exploratory and confirmatory factor analyses were conducted with sample sizes of 200 and 182, respectively. Reliability was assessed using Cronbach's alpha coefficient, MacDonald's omega, and intra-class correlation coefficient.

**Results:**

The exploratory factor analysis resulted in the exclusion of four items, leaving a final selection of 15 items. These items were categorized into three factors: classmates, clinical staff, and inclusive educational environment, which accounted for 49.16% of the overall variance. The confirmatory factor analysis indicated that the model was a good fit for the observed data, and the subscales had high internal consistency (Cronbach's alpha coefficient was 0.752 to 0.880) and stability (intra-class correlation coefficient was 0.889 to 0.968).

**Conclusion:**

According to the results, it can be concluded that the Persian version of the SBNS scale demonstrates sufficient validity and reliability in assessing students' sense of belonging to the nursing school.

**Supplementary Information:**

The online version contains supplementary material available at 10.1186/s12912-024-01738-x.

## Background

Nurses constitute the largest cohort of healthcare professionals, serving as integral and indispensable contributors to the enhancement of individual and societal well-being [1]. One of the primary strategies for addressing the shortage of nursing staff involves prioritizing the retention of the current nursing workforce [2]. The pressing demand for nurses necessitates that nursing schools and clinical staff establish inclusive environments to enhance nursing students' sense of belonging, thereby fostering their retention and successful graduation [3].

Belonging refers to the experience of being accepted, included, and valued within a particular context or group [4]. According to Hagerty et al. (1992), a person's experience of involvement in a system or environment leads them to believe they are an essential component of that system or environment [5]. The sense of belonging within the context of clinical education is a profoundly personal encounter that is connected to an individual's subjective perception in three key aspects. These aspects include a) a sense of safety, acceptance, appreciation, and respect from a specific group, b) proficient communication with said group, and c) aligning one's values with the professional values upheld by the group [6]. Strayhorn (2012) defines the sense of belonging in education as the social support students perceive in the university environment, the feeling or experience of engagement, and the feeling of being essential or welcomed, respected, and valued [7].

The sense of belonging among students is positively correlated with several aspects of their university experiences. For instance, the sense of belonging enhances and facilitates favorable educational experiences [8–11], student-faculty connections [12], self-worth [13], self-assurance, motivation [8, 14], job contentment [15–18], proficiency [19], students' aspirations for career advancement in their chosen field, and student retention [15, 20–22], all of which are generally associated with their academic advancement and overall success at the university [23]. Multiple research studies have demonstrated that students who possess a heightened sense of belonging exhibit a greater emphasis on the value of learning and display increased motivation toward achieving academic success [24]. Furthermore, it has been observed that nursing students who have positive learning experiences and are satisfied with their studies [14, 25] tend to develop a sense of belonging. This sense of belonging is also associated with higher self-esteem [14], effective interaction with colleagues [26], increased motivation to learn and stay in the nursing profession [27], and reduced likelihood of leaving the job [28]. Additionally, it is worth noting that a sense of belonging is inversely related to perceived stress [14] and the disrespect of nurses [29]. Therefore, fostering a sense of belonging is considered one of the most crucial needs for nursing students in the clinical environment [3].

To date, a range of tools has been developed to assess the sense of belonging. Two commonly used tools in this context are the Belongingness Scale-Clinical Placement Experience (BES-CPE) developed by Levett-Jones et al. [16] and the General Belonging Scale (GBS) developed by Malone et al. [30]. The GBS was the first tool developed to differentiate between the concept of belongingness and the need for belonging. This tool assesses various levels of belonging, encompassing familial, close friendships, and community connections, as well as a broader sense of belonging that extends beyond interpersonal relationships [30]. Levett-Jones et al. (2009) developed the BES-CPE tool to assess students' perception of their sense of belonging in the clinical work environment. This tool consists of three microscales: self-esteem, group cohesion, and efficiency [16]. However, these instruments primarily assess the perception of belongingness within the context of family, social groups, and work environments. Prior to the tools mentioned above, there was a lack of a comprehensive scale to assess the level of belonging experienced by nursing students in various settings such as the classroom, clinical environment, peer groups, and other environments that contribute to fostering a sense of belonging [12, 31].

To assess, quantify, and determine the factors contributing to the enhancement of nursing students' sense of belonging, it is crucial to utilize a well-defined and all-encompassing scale. The newest instrument to measure nursing students' sense of belonging is the Sense of Belonging in Nursing School (SBNS), developed by Patel et al. in the English language in 2022. The tool consists of 19 items and focuses on students' experiences in four distinct areas: the clinical environment with instructors, the clinical environment with nursing personnel, the classroom environment with professors, and interactions with other nursing students [32]. Given that there has been no psychometric testing of this instrument in other countries, and considering the absence of a valid scale to assess the sense of belonging, particularly among nursing students in Iran, it is essential to conduct a psychometric evaluation of this scale. Hence, the objective of this study was to assess the validity of the Persian version of the SBNS scale. The validation of this tool is anticipated to facilitate its utilization in future research endeavors aimed at assessing and identifying potential ways of improving the sense of belonging among nursing students, thereby effectively contributing to their overall growth and development.

## Methods

### Study design

The present study employed a quantitative and cross-sectional design. It was conducted on nursing students from Shahroud and Semnan Universities of Medical Sciences. Data were collected using the convenience sampling method from 3/6/2023 to 24/8/2023.

### Scale

The original version of SBNS was developed by Patel et al. (2022). This scale is made up of 19 items. The items were scored on a five-point Likert scale, with the options "Strongly disagree" (1 point), "Disagree" (2 points), "Neutral" (3 points), "Agree" (4 points), and "Strongly agree" (5 points). SBNS is divided into four subscales: clinical personnel (items 1 to 6), clinical instructors (items 7 to 8), classroom (items 9 to 12), and classmates (items 13 to 19). The SBNS instrument scores range from 19 to 95, with a higher score indicating a stronger sense of belonging [32].

### Translation

Following email correspondence and receiving explicit permission from the original designer, Professor Patel, the translation of the tool was conducted in accordance with the translation process established by the World Health Organization (WHO) [33]. The process involved the following steps:

- *Initial translation*: The scale was initially translated from English to Persian by two separate translators. One translator had a doctorate in nursing and was fluent in English, while the other had a doctorate in English language and literature.
- *Forward–backward translation*: The translated version was then translated back into English by two proficient individuals to ensure accuracy and consistency.
- *Optimal translation*: The best translation was selected after comparing the initial and back-translated versions, taking into account the optimal translation process.
- *Cross-referencing*: The revised version was cross-referenced with the original English version by the study team to ensure that the translated version accurately represented the original content.
- *Finalization*: The finalized version was then submitted to Professor Patel for her final endorsement, which was duly granted.
- *Incorporation of recommendations*: All of the recommendations were incorporated into the ultimate iteration of this measurement tool to ensure its accuracy and reliability [34, 35].

### Face validity

The current stage of the evaluation consisted of two main components: a qualitative and a quantitative face validity assessment. To begin with, a qualitative face validity evaluation was conducted by conducting face-to-face interviews with ten nursing students. These interviews aimed to gather the students' views and insights regarding the appropriateness, difficulty, relevance, and clarity of the subject matter.

To assess the quantitative face validity, a group of ten nursing students was requested to evaluate the importance of each question on a 5-point Likert scale. The scale was designed to measure the significance of the questions, with ratings ranging from 'very important' (scored as 5) to 'not important' (scored as 1). To calculate the impact score of the items, the formula 'Impact score = Frequency (%) × Importance was used.

The concept of frequency, represented as a percentage, refers to the proportion of individuals who assign a rating of 4 or 5 points to the item. On the other hand, the purpose of assessing importance is to determine the average importance score using the Likert scale. The grading procedure involves evaluating 1.5 criteria, which are determined by calculating the average of three factors and considering a frequency threshold of 50%. If the resulting impact score exceeds 1.5, the item is considered appropriate for further analysis and will be retained. However, items that have an impact score below 1.5 will be kept for revision and modification [36, 37].

### Content validity

Similar to the previous section, an evaluation of content validity was conducted using both qualitative and quantitative methods. In order to qualitatively assess the content validity, an interview was conducted with 12 experts (consisting of ten experts in the field of nursing and two experts in scale development). The purpose of the interview was to observe the grammar, appropriate expressions, item placement, and accurate scoring of the scale. After considering the feedback received, we made modifications to items 9 to 11 in the Persian version to enhance clarity.

The following phase involved assessing the quantitative content validity by estimating the content validity ratio (CVR) and content validity index (CVI) for the items. In this regard, the aforementioned experts were asked to evaluate the significance of each item based on criteria such as 'not necessary' (scored as 1), 'useful but not necessary' (scored as 2), and 'necessary' (scored as 3) [38]. The CVR was calculated using the formula: CVR = (ne—[N / 2]) / (N / 2), where 'N' represents the total number of experts and 'ne' represents the number of experts who deemed the item as 'necessary'. According to the Lawshe table, the minimum acceptable CVR is 0.56, considering a panel of 12 experts [39].

The assessment of CVI was based on the opinions of the expert panel. CVI measures the degree of relevance of the scale items to the overall concept of the scale. The experts reviewed and scored each item based on the options 'not relevant = 1', 'somewhat relevant = 2', 'relevant but needs revision = 3', and 'completely relevant = 4'.

The CVI for each item was calculated by dividing the number of experts who rated the item as 3 or 4 by the total number of experts. Items with a CVI score higher than 0.79 were considered acceptable, while those with scores between 0.70 and 0.79 were deemed questionable and subject to revision. Items with scores below 0.70 were considered unacceptable [40].

The scale content validity index (S-CVI) and scale content validity ratio (S-CVR) were calculated by averaging the CVI and CVR values respectively. An S-CVI greater than 0.9 is considered acceptable [41]. Additionally, each item was evaluated using the modified Kappa statistic (K*) to assess chance agreement among the expert panel. Items with a K* of 0.7 or higher were considered adequate [42].

### Participants and the study setting

Following Munro's guidelines [43], we decided to select a sample of 5–10 nursing students per item for both exploratory factor analysis (EFA) and confirmatory factor analysis (CFA). To obtain the sample, a total of 382 nursing students from Shahroud and Semnan Universities of Medical Sciences were chosen using specific entry criteria and the census sampling method. The inclusion criteria for this study included being enrolled in the second semester or beyond of a nursing program, not having any diagnosed mental disorders, and not currently taking any neuroleptic medications. These criteria were self-reported or confirmed by a medical professional or university psychologist. On the other hand, students who had been expelled or transferred to other educational institutions and therefore couldn't participate were excluded from the study.

### Construct validity

The construct validity of SBNS was assessed using maximum likelihood exploratory factor analysis (MLEFA) with Promax rotation on the initial set of 200 responses for EFA. Sampling adequacy was evaluated using the Kaiser–Meyer–Olkin (KMO) and Bartlett's tests. KMO values ranging between 0.7 and 0.8 were considered good, while values between 0.8 and 0.9 were considered excellent [44]. The inclusion of an item in a latent factor was determined based on its factor loading, which was approximately 0.33, estimated using the formula: CV = 5.152 ÷ √ (*n* – 2); Here, CV represents the critical value, and n is the sample size [45]. In general, a factor loading above 0.3 is considered acceptable [46]. Subsequently, items with a loading below 0.3 were removed from the EFA. However, some researchers suggest that factor loadings of 0.4 or higher are more appropriate. It is essential to consider the context and the specific scale being used, as the acceptable range for factor loading may vary depending on the study and the theoretical framework. However, the acceptable strength of the factor loading depends on the theoretically assumed relationship between the item and the factor.

Confirmatory factor analysis (CFA) was then conducted to assess the goodness-of-fit and align the proposed model with the actual model in the study population. In other words, CFA aimed to validate the model based on the EFA findings. Various fit indices were utilized, including the root mean square error of approximation (RMSEA) < 0.08, comparative fit index (CFI) > 0.9, parsimony comparative fit index (PCFI) > 0.5, parsimony normed fit index (PNFI) < 0.5, incremental fit index (IFI) > 0.9, and CMIN / DF > 3, to assess the model fit [47].

### Convergent and discriminant validity

To assess the convergent and discriminant validity of SBNS from Fornell and Larcker's perspective, several metrics were examined including the average extracted variance (AVE), maximum shared squared variance (MSV), and composite reliability (CR). An AVE value greater than 0.5 or a CR value greater than 0.7 is generally regarded as indicative of appropriate convergent validity. Additionally, if the AVE value is higher than the MSV value, it confirms the instrument's discriminant validity [48]. AVE is commonly used as an accurate measure of convergent validity. Additionally, a composite reliability (CR) value greater than 0.7 is often employed to assess convergent validity in psychological studies [49].

### Reliability

To assess the internal consistency of SBNS, Cronbach's alpha, and McDonald's omega coefficients were calculated for each extracted factor. A minimum threshold of 0.7 was set for both coefficients to indicate high internal consistency. Furthermore, the construct reliability (CR) of each factor was examined, with CR scores above 0.7 indicating good reliability [50].

The stability of SBNS was assessed using intra-class correlation coefficients (ICC). A minimum acceptable ICC value of 0.75 was determined as the threshold [51]. To evaluate stability, a group of 30 nursing students completed the scale twice, with a two-week interval between the administrations.

### Normality, outliers, and missing data

Distribution charts and Mahalanobis distance (*p* < 0.001) were utilized to assess both univariate and multivariate outliers. Additionally, we conducted an investigation into the univariate and multivariate normality distribution, taking into account the skewness (values within ± 3), kurtosis (values within ± 7), and the Mardia coefficient < 8 [52]. The data from this study did not exhibit a significant departure from the normal distribution. A listwise missing procedure was employed to estimate CFA. Listwise deletion was chosen as the preferred method over imputation due to the observation that non-response was linked to incomplete questionnaires and non-response [53]. Statistical analysis was conducted using SPSS and AMOS version 26.0.

### Ethical approval and consent to participate

The Ethics Council in Biomedical Research of Shahroud University of Medical Sciences approved the current study (IR.SHMU.REC.1402.029). At the beginning of the research, the goals and conditions of participation in the study were announced to the participants. The authors adhered to the Committee on Publication Ethics (COPE) principles in publishing their findings. Informed consent was obtained from all participants.

## Results

A comprehensive sample size of 382 undergraduate nursing students was involved in the present study. Out of the total, 199 individuals (52.1%) were identified as women, 358 individuals (93.7%) were reported as single, and 187 individuals (49%) were recorded as residents of student dormitories. The demographic information of the participants is presented in Table 1.

**Table 1:** The characteristics of study participants (*n* = 382)

Variables	Number (%)
**Gender**	
Male	183 (47.9)
Female	199 (52.1)
**Marital Status**	
Single	358 (93.7)
Married	22 (5.8)
Divorced	2 (0.5)
**Residence Status**	
Student dormitory	187 (49)
Rental House	25 (6.5)
With family	170 (44.5)
	Mean (SD)
**Age (years)**	21.76 (1.63)
**Interest in nursing (up to 10)**	6.81 (2.20)

*n* Frequency, *SD* Standard deviation

### Face and content validity

The results of the face validity assessment indicated that all items of the tool were deemed appropriate, clear, and important. Additionally, the quantitative face validity results showed that all scores exceeded the threshold of 1.5.

Based on the recommendations of 12 experts, some items were updated in terms of qualitative content validity. In quantitative content validity, the content validity ratio (CVR) and content validity index (CVI) were calculated for each item. None of the items were eliminated when using the appropriate cutoff value of 0.56. The scale-level content validity ratio (S-CVR) and scale-level content validity index (S-CVI) were calculated to be 0.82 and 0.98, respectively. Furthermore, the modified Kappa statistic for all items was satisfactory, exceeding the threshold of 0.7.

### Construct validity

During the maximum likelihood exploratory factor analysis (MLEFA), the Kaiser–Meyer–Olkin (KMO) value was found to be 0.854, indicating a good level of sampling adequacy. Additionally, Bartlett's test of sphericity yielded a significant value of 2187.381 (*P* < 0.001), suggesting that the correlations between variables were sufficiently large for factor analysis.

The MLEFA model extracted three factors, which were determined based on eigenvalues greater than one. The results, as presented in Table 2, reveal that these three factors together accounted for 49.16% of the total variance.

**Table 2:** Exploratory Factors analysis of the SBNS (*N* = 200)

Factors	Qn. Item	Factor Loading	h^2^	λ	%Variance
**Classmate**	15: I am comfortable with my classmates	0.873	0.727	3.376	24.91
	16: If needed, my classmates are available to help me	0.814	0.655		
	13: I have a strong bond with other classmates	0.797	0.631		
	18: My classmates respect me	0.688	0.490		
	14: If I miss a class, my classmates will follow up on my situation	0.655	0.447		
	19: My classmates accept me	0.641	0.462		
**Clinical staff**	3: The nursing staff respect me as a student	0.748	0.516	1.897	12.65
	5: As a nursing student, I am welcome in the academic environment	0.670	0.453		
	4: The nurse shares the necessary information with me about patient care	0.617	0.388		
	6: Nursing staff include me in their conversations during clinical care	0.577	0.386		
	2: I contribute to the care of patients	0.415	0.206		
**Inclusive educational environment**	12: The faculty supports my learning	0.813	0.639	1.740	11.60
	11: I trust the academic faculty members in my academic counseling	0.721	0.493		
	9: The faculty provides an inclusive environment (providing teaching and learning opportunities)	0.663	0.503		
	10: Despite my concerns, I can easily attend college	0.343	0.213		

*Abbreviations:* *h*^2^ Item Communalities, *λ* Eigenvalue

Furthermore, four items (1, 7, 8, and 17) from the original version of the tool were removed due to their factor loadings falling below the threshold of 0.3. Consequently, the total number of scale items for analysis was reduced to 15 (Additional file 1).

### Confirmatory factor analysis

**Fig. 1:**
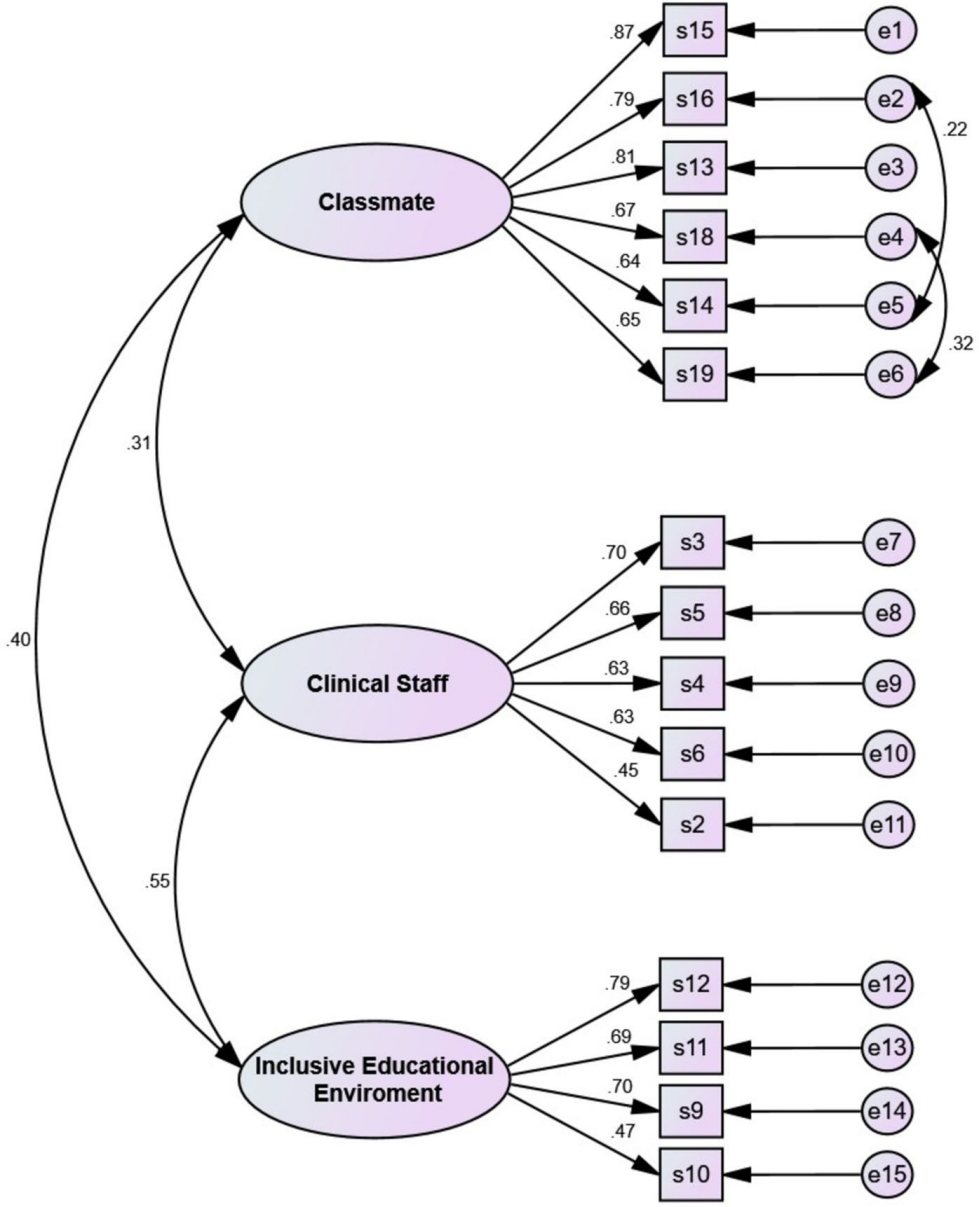
The final model of the SBNS based on CFA (*N* = 182)

CFA findings confirmed all goodness of fit indices of the final model (χ^2^ = 165.065; DF = 85, *P* < 0.001, CMIN/DF = 1.94, PCFI = 0.779, PNFI = 0.749, RMSEA = 0.05 (CI 90%: 0.03, 0.06), IFI = 0.963, CFI = 0.962, GFI = 0.947, AGFI = 0.925 and PGFI = 0.671 (Fig. 1).

### Convergent and discriminant validity

In terms of assessing convergent validity, it was found that only the average extracted variance (AVE) for factor 1 exceeded the threshold of 0.5. However, the AVE values for factor 2 (0.386) and factor 3 (0.453) were slightly below the 0.5 threshold, as indicated in Table 3. Considering the CR and maximum reliability (MaxR) values, it can be concluded that convergent validity was achieved for all three factors. Furthermore, as the AVE values were higher than the maximum shared squared variance (MSV) for each factor, it confirms the presence of discriminant validity.

**Table 3:** Convergent and Discriminant Validity, and Reliability of the SBNS

Factors	CR	AVE	MSV	MaxR (H)	α	Ω	ICC
Classmate	0.879	0.551	0.157	0.897	0.880	0.886	0.968
Clinical staff	0.755	0.386	0.302	0.769	0.753	0.765	0.900
Inclusive educational environment	0.763	0.453	0.302	0.792	0.752	0.763	0.889

*Abbreviations*:*SBNS* Sense of Belonging in Nursing School, *CR* Composite Reliability, *AVE* Average Variance Extracted, *MSV* Maximum Shared Squared Variance, *α* Cronbach's alpha, *Ω* McDonald's omega, *ICC* Intraclass Correlation Coefficients

### Reliability

Cronbach's alpha, McDonald's omega, and Intra-class correlation coefficients of three factors extracted from SBNS were adequate (Table 3). The ICC of all items was estimated equal to 0.960 (CI 95%: 0.981–0.917). Also, CR higher that 0.7 indicated adequate construct reliability.

## Discussion

Based on the findings of the current study, it has been determined that the SBNS scale is composed of three distinct factors: "classmates," "clinical staff," and "an inclusive educational environment." These 15 items within the scale predict a noteworthy percentage of the total variance. The SBNS scale originally had 19 items and a four-factor structure (1- Classmates/Cohort, 2- Classroom, 3- Clinical-Staff, and 4- Clinical-Instructors) [32]. The first factor identified in the Persian version of the SBNS was the "classmates" encompassing six items primarily associated with fostering a sense of belonging within the college educational environment through interactions with classmates. This element accounted for the highest proportion of sense of belonging and demonstrated its significance in nursing school sense of belonging. This factor consists of six items, each with a positive expression. Patel et al. (2022) recognized Classmates/Cohort as one of four factors in the psychometrics of the early edition of SBNS, which is equivalent to the present factor in the Persian version [32]. They are critical for instilling a strong sense of "being a part of the community" in classmates, which supports members' effect on one another, including mutual trust [54]. It is important to remember that the presence of classmates with whom a nursing student may readily converse about his education is a form of social support in the nursing school setting. In this regard, one of the components known to increase academic self-efficacy in nursing students is social support [55].

The "clinical staff" emerged as the second factor in the current version, encompassing five items. This factor primarily focuses on student engagement in meaningful scientific discussions about patient care, active involvement in patient care responsibilities, and the regard and dignity shown to nursing students by the clinical staff. One of the detected factors in the initial edition of this scale was clinical staff, which is the same factor observed in the Persian version [32]. In this regard, it should be noted that clinical staff support for students during their educational rounds is a protective factor to improve their sense of belonging, as demonstrated by the findings of a study conducted by Lopez et al. (2018) that clinical staff support nursing students is a protective factor to prevent the intention to drop out of nursing education [56]. Furthermore, it is conceivable that the clinical staff does not encourage students and displays violence or rudeness against them. In this regard, the findings of Patel et al. (2022) revealed that clinical staff rudeness toward students has a negative and significant link with nursing students' sense of belonging [29].

The last factor was also identified as the inclusive educational environment. The items associated with this factor pertain to the support provided by the nursing faculty by means of a conducive learning environment and academic staff members, such as academic counseling. This factor corresponds precisely to the classroom factor in the first iteration of SBNS [32]. Consequently, instructors' support and communication with nursing students are crucial factors that affect students' academic performance [57]. In addition, Tharani et al. (2017) discovered that nursing students believed that the quality of their "educational environment" had a significant impact on their mental health. In addition, they cited the role of professors, teaching methods, academic expectations, and the availability of learning resources as other important factors affecting students' psychological well-being and academic performance [58].

Based on the results of the current study, all the fit indices in the CFA were in the acceptable range, so the model fits well with the data. The original version of the SBNS scale and a similar instrument designed by Levett-Jones et al. (2009) to measure nursing students' sense of belonging were not evaluated using CFA [16, 32]. In contrast, Ashktorab et al. (2015) evaluated the Persian version of the Belongingness Scale—Clinical Placement Experience (BES-CPE) and demonstrated, using CFA, that the model is well-fitting [59]. In addition, the CFA results from the study conducted by Kim and Jung (2012) in conjunction with the validation of the Korean version of the BES-CPE demonstrated that the tool model is a good fit with three factors (self-esteem, connectedness, and efficacy) [60].

The present study shows that SBNS items in the final model have good convergent and discriminant validity. The original version of SBNS was not evaluated in this respect [32]. However, Kim and Jung (2012), in a similar study, measured the validity of the Korean version of the BES-CPE and found that SBNS has a positive and significant correlation with self-esteem and self-directed learning [60]. Also, Daniels et al.'s (2020) study showed that the original belongingness scale has acceptable convergent validity. The sense of belonging is correlated with students' overall satisfaction with the undergraduate course [61].

In this study, the Cronbach's alpha coefficients for all components of the Persian version of the SBNS were found to be acceptable, demonstrating strong internal consistency among the scale items. Additionally, the construct reliability (CR) of the scale was evaluated through confirmatory factor analysis (CFA), revealing a sufficient level of construct reliability. One of the benefits of CR measurement is that it is independent of the number of scale items and sample size [62]. In Patel et al.'s study (2022), Cronbach's alpha coefficients for the entire scale and its subscales, including classmates, classroom, clinical staff, and clinical instructors, all surpassed the 0.9 threshold, consistent with the findings observed in the present study [32]. Also, Cronbach's alpha values for the Persian version of the BES-CPE were calculated by Ashktorab et al. (2015); the total score was 0.92, and the subscales ranged from 0.80 to 0.85 [59]. In addition, Cronbach's alpha values for the original BES-CPE total score were reported to be 0.92 (subscales between 0.80 and 0.92) [16].

The assessment of SBNS measurement stability was conducted using test–retest analysis. The findings indicated a substantial association between the initial and subsequent assessments. These results supported the scale's excellent repeatability, demonstrating that the Persian version of the SBNS scale exhibits appropriate stability based on intra-class correlation (ICC). It's noteworthy that the stability of this scale was not reported in its original version [32]. In a similar study, Ashktorab et al. (2015) calculated the ICC value of the Persian version of the BES-CPE for the whole scale as 0.95 [59].

There are now just 15 items in the SBNS Persian translation, as items 1, 7, 8, and 17 have been eliminated. A higher overall score (between 15 and 75) indicates a stronger sense of belonging within the nursing school. The Persian version of the SBNS comprises three factors: classmates (scores of six to 30), clinical staff (scores of five to 25), and inclusive educational environment (scores of four to 20).

Since only two nursing institutions participated in the data collection, the generalizability of the study may be limited. Additionally, this scale must be adapted to the culture of other Persian-speaking nations. Additionally, as the SBNS scale relies on self-reported measures, there is a potential for response bias to impact the results.

## Conclusions

The present study revealed that the SBNS scale consists of 15 items and three factors in Iranian nursing students, which predicts approximately 50% of the variance in nursing school belongingness. This scale has high levels of reliability, internal consistency, and construct validity for measuring the sense of belonging in nursing school. In addition, the SBNS scale can be utilized as a practical instrument in nursing education to solve nursing student problems.

### Implications for nursing education

This scale can be used to evaluate and improve the sense of belonging for nursing students, which is essential for their motivation and the quality of patient care. By understanding the factors that contribute to a strong sense of belonging, nursing educators can implement targeted interventions to create a more inclusive and supportive educational environment for their students. The general importance of such studies lies in the potential for cross-cultural comparisons and the advancement of nursing research and practice on a global scale. By validating the Persian version of the SBNS scale, this study contributes to the availability of a reliable measurement tool for assessing the sense of belonging among Persian-speaking nursing students, which in turn can facilitate comparative research across different cultural and linguistic contexts. Additionally, the rigorous translation and psychometric evaluation process followed in this study can serve as a valuable model for similar efforts in other languages, thereby enhancing the cross-cultural applicability and generalizability of nursing research instruments.

### Supplementary Information

**Additional file 1. **

## Data Availability

The datasets used and/or analyzed during the current study are available from the corresponding author on reasonable request.

## Abbreviations

CVR: Content validity ratio
CVI: Content validity index
I-CVI: Item content validity index
S-CVI: Scale content validity index
EFA: Exploratory factor analysis
CFA: Confirmatory factor analysis
ML: Maximum Likelihood
KMO: Kaiser–Meyer–Olkin
RMSEA: Root Mean Square of Error of Approximation
CFI: Comparative Fit Index
PNFI: Parsimonious Normed Fit Index
IFI: Incremental Fit Index
ICC: Intra-class correlation coefficient
PCFI: Parsimony Comparative Fit Index
BES-CPE: Belongingness Scale-Clinical Placement Experience
GBS: General Belonging Scale
SBNS: Sense of Belonging in Nursing School
WHO: World Health Organization
AVE: Average Extracted Variance
MSV: Maximum Shared Squared Variance
CR: Composite Reliability
