# Supplementary material for: Psychometric evaluation of the Persian version of the Sense of Belonging in Nursing School (SBNS): a quantitative and cross-sectional design
Source: BMC Nurs. 2024 Jan 27;23:73. doi: 10.1186/s12912-024-01738-x (PMC10822173; doi:10.1186/s12912-024-01738-x)
Supplement: Supplementary file 1 — Additional file 1. [file 12912_2024_1738_MOESM1_ESM.pdf]

The final version of the Persian version of the Sense of Belonging in Nursing School scale (SBNS) (15 items)

Item	Strongly disagree	Disagree	Neutral	Agree	Strongly agree
2- I contribute to the care of patients.					
3- The nursing staff respect me as a student.					
4- The nurse shares the necessary information with me about patient care.					
5- As a nursing student, I am welcome in the academic environment.					
6- Nursing staff include me in their conversations during clinical care.					
9- The faculty provides an inclusive environment (providing teaching and learning opportunities).					
10- Despite my concerns, I can easily attend college.					
11- I trust the academic faculty members in my academic counseling.					
12- The faculty supports my learning.					
13- I have a strong bond with other classmates.					
14- If I miss a class, my classmates will follow up on my situation.					
15- I am comfortable with my classmates.					
16- If needed, my classmates are available to help me.					
18- My classmates respect me.					
19- My classmates accept me.					
